# A survey of management practices on Irish dairy farms with emphasis on risk factors for Johne’s disease transmission

**DOI:** 10.1186/s13620-014-0027-9

**Published:** 2014-12-24

**Authors:** Aideen E Kennedy, Eugene F O’Doherty, Noel Byrne, Jim O’Mahony, E M Kennedy, Riona G Sayers

**Affiliations:** Animal & Bioscience Research Department, Animal & Grassland Research and Innovation Centre, Teagasc, Moorepark, Fermoy, Co. Cork, Ireland; Department of Biological Sciences, Cork Institute of Technology, Bishopstown, Co. Cork, Ireland

**Keywords:** Johne’s disease, Survey, Management practices, Biosecurity

## Abstract

**Background:**

Johne’s disease (JD) is a chronic granulomatous enteritis affecting ruminants. A number of farm management practices are associated with increased risk of JD transmission. The aim of the current study was to document JD-related management practices currently employed on Irish dairy farms.

Survey questions focused on calving area (CA), calf and manure management. Independent variables (region, calving-season, enterprise type, herd size and biosecurity status) were used to examine influences on JD associated dependent variables (survey questions). Additionally general biosecurity practices were also examined.

**Results:**

Results showed management practices implemented by Irish dairy farmers pose a high risk of JD transmission. Of the farmers surveyed, 97% used the CA for more than one calving, 73.5% and 87.8% pooled colostrum and milk respectively, 33.7% never cleaned the CA between calving’s, and 56.6% used the CA for isolating sick cows. Survey results also highlighted that larger herds were more likely to engage in high risk practices for JD transmission, such as pooling colostrum (OR 4.8) and overcrowding the CA (OR 7.8). Larger herds were also less likely than smaller herds to clean the CA (OR 0.28), a practice also considered of risk in the transmission of JD.

**Conclusion:**

Many management practices associated with risk of JD transmission were commonly applied on Irish dairy farms. Larger herds were more likely to engage in high risk practices for JD transmission. Control programmes should incorporate educational tools outlining the pathogenesis and transmission of JD to highlight the risks associated with implementing certain management practices with regard to JD transmission.

**Electronic supplementary material:**

The online version of this article (doi:10.1186/s13620-014-0027-9) contains supplementary material, which is available to authorized users.

## Background

Johne’s disease (JD), a chronic granulomatous enteritis of ruminants, is caused by the bacterium *Mycobacterium avium* subspecies *paratuberculosis* (MAP) [1]. Significant economic losses have been reported on cattle farms due to infection with MAP. Such losses are primarily due to decreased slaughter value [2], reductions in milk production in dairy cows [3], sub-optimal fertility [4], and an increase in cow replacement costs [5]. The impact of JD on animal health and on-farm profitability has led to considerable interest in the control of MAP at farm level. Controlling JD however proves difficult due to the variable progression from sub-clinical to clinical stages of disease, combined with diagnostic difficulties especially in the early stages of infection [6]. As test and cull programmes alone prove largely ineffective in eradicating MAP from a herd [7], incorporation of improved calf management practices, including calf-related hygiene, may prove of more benefit in reducing on farm prevalence [8].

Infection with MAP predominantly occurs in calves, with animals less than six months of age being most susceptible [9]. The severity and rate of JD progression in individual animals are dependent on the MAP exposure dose and the age of the animal at infection [10]. Infection usually occurs via the faecal-oral route, although in-utero transmission can occur [11]. Doré E, Paré J, Côté G, Buczinski S, Labrecque O, Roy J and Fecteau G [12], concluded that exposure of calves to adult faeces is the most important risk factor in MAP transmission. Faecal-oral transmission is facilitated by faecal contamination of a calf’s environment and feedstuffs, with the primary environmental risk factors for neonatal infection being faecal contamination of the udder or calving pens [13]. Colostrum and milk from infected cows can also contain quantities of MAP capable of infecting calves [14,15]. Feeding of pooled colostrum from multiple cows [14], and feeding milk containing antibiotic residues to calves [16], are also both considered to increase the risk of MAP infection within a herd.

Additional management-related risk factors for MAP transmission include group housing of periparturient cows [17], the presence of more than one cow in a calving pen [18], use of group calving pens [19], faecal contamination of udders of periparturient cows [20], and use of maternity pens that are not cleaned between each calving [21]. Larger sized herds [17,22-24], are associated with an increased risk of testing MAP ELISA positive. Allowing young-stock access to pasture contaminated with adult manure can also be considered a risk factor due to the prolonged survival of MAP in slurry [25]. Finally, biosecurity [26,27], is an essential component of disease prevention in general, and is equally important in the prevention of JD, with purchase of animals considered a significant route of MAP transmission between farms [28].

Concern has been raised regarding the zoonotic potential of MAP [29] a potential link between MAP and Crohn’s disease in humans having been postulated. Proof of a causal link would have important consequences for the global dairy industry [30]. The possible public health implications of MAP make it incumbent on milk producing nations to minimise the risk of consumers ingesting MAP contaminated milk. The most recent estimate of JD herd exposure prevalence in Irish cattle is approximately 20% [31], which compares favourably with estimates in other European countries [32]. Additionally, between the years of 1995 and 2002 only 232 clinically infected animals (an average of approximately 30 animals per year in a cattle population of approximately six million) were detected by the Irish Department of Agriculture, Food and the Marine laboratories (DAFM) [33]. Although a relatively low prevalence is reported, the dairy industry plays a critical role in Ireland’s economy [34] and as such a JD pilot control programme has been embarked upon to further reduce the levels of MAP in Irish cattle. This Animal Health Ireland (AHI) [35] co-ordinated programme uses risk assessment and management plans (RAMPs) as an integral part of the scheme [1]. These risk assessments involve evaluation of four key JD risk areas namely, management of pre-weaned heifers, management of heifers to first calving, mature cow environment and hygiene, and management of the calving area.

Investigations into herd demographics [36] and risk factors associated with introduction and transmission of JD and testing JD positive on Irish dairy farms have previously been conducted [37,38]. The risk factors identified in these studies included larger herd size [38], importation of cattle from abroad [36,38], and not using individual calving pens [37]. These findings are in agreement with the international studies described previously. Although risk factors for testing positive for MAP have been identified in Ireland, a national survey documenting the prevalence of application of JD risk-associated management practices at farm level has not previously been reported. Such a study may highlight underlying reasons for Ireland’s relatively low prevalence of JD test positive individuals and herds. The aim of the current study, therefore, was to document utilisation of management factors associated with JD transmission on Irish dairy farms, based on both national and international risk data, using a geographically representative group of Irish dairy farms. This will provide a baseline for JD risk in Ireland, which can subsequently be used to allow targeting of specific management practices that require improvement as part of control programmes. Key influences on the application of JD-associated management factors were also investigated.

## Methods

### Survey procedure

The survey was conducted as a postal survey with survey packs containing a cover letter, a self-addressed envelope, and a questionnaire, mailed to participants for completion and return. The study population included farmers that participated in a larger disease prevalence study, the selection of whom has previously been outlined by O’Doherty E, Sayers R and O’Grady L [39]. In brief, 500 randomly selected members of HerdPlus® (a breeding management decision support tool co-ordinated by the Irish Cattle Breeding Federation [ICBF]) were invited to participate. Selection was based on stratified proportional sampling using strata of herd size and geographical location. A total of 312 herds elected to participate in the study with participation entirely voluntary and non-incentivised. The study population has previously been shown to be geographically representative of Irish dairy herds [39]. The overall project was approved by the Moorepark ethics committee in November 2008.

### Survey questionnaire

**Figure 1:**
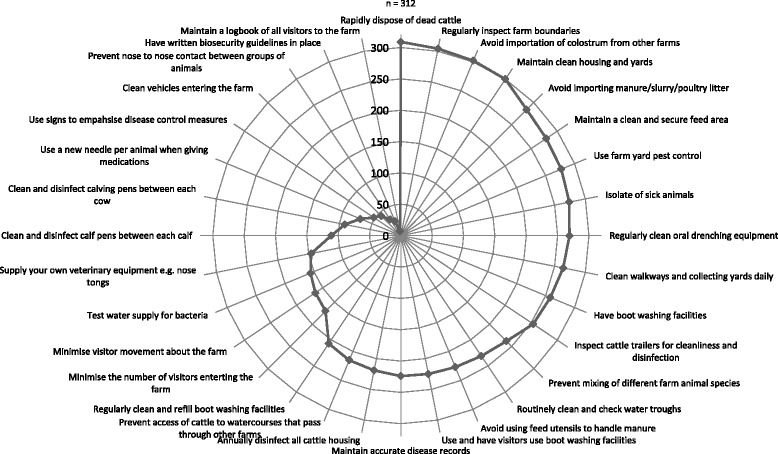
**Responses to biosecurity survey questions.** The level of implementation of biosecurity practices are listed in descending order from the 12 o’ clock position (n = 312).

Questions were compiled based on information gathered from peer-reviewed publications, a commercially available web-based herd-health management tool [2], and Teagasc researcher experience of Irish dairying systems. Following consultation with researchers at the Animal and Grassland Research and Innovation Centre, Teagasc (Irish Agriculture and Food Development Authority) and piloting of the questionnaire by farm managers based at seven Teagasc research farms, a number of minor modifications were made to the questionnaire prior to circulation to study participants. The final questionnaire consisted of an initial section containing 17 JD-associated questions (Table 1) and a second section containing a further 30 questions examining general bioexclusion and biocontainment (collectively referred to as biosecurity) management practices (Figure 1). Johne’s disease associated questions related to the calving-area (CA) and CA hygiene, milk and colostrum management, and access of young calves and in-calf heifers to adult faeces. These survey questions (dependent variables) were presented in a closed format with three response options offered, namely Yes (Y), No (N), or Sometimes (S). A subset of the population (approximately 10%) was re- surveyed in order to quantify the Sometimes responses. Where Sometimes was chosen as an answer, an extra closed question was asked with the options of either A = <50% of the time or B = >50% of the time offered (Table 1). Biosecurity-related questions were again presented as closed questions offering Yes and No binary responses.

**Table 1:** **JD Questionnaire responses**

**Que.**	**Management variable**	***n***	**Response**	**Outcome (%)**	If sometimes chosen- what % of the time?*	
Calving area (CA) management					A: Less than 50%	B: More than 50%
1	Is the CA frequently used for more than one calving at any one time?	303	No	3		
			Sometimes	27	x	
			Yes	70		
2	Is the CA overcrowded? (e.g. more than five cows in calving pen at any one time)	302	No	57.6		
			Sometimes	29.1	X	
			Yes	13.2		
3	Is the CA cleaned out between every calving and bedded with clean dry bedding?	300	No	33.7		
			Sometimes	37	X	
			Yes	29.3		
4	Is the CA used to house sick cows?	297	No	42.4		
			Sometimes	54.9	X	
			Yes	2.7		
5	Do cows have manure soiled legs and udders?	300	No	43.3		
			Sometimes	51	X	
			Yes	5.7		
Calf feeding management						
6	Do new born calves stay with mother in CA for more than six hours?	304	No	17.1		
			Sometimes	39.5	X	
			Yes	43.4		
7	Is the calf allowed to suckle from the cow?	303	No	7.3		
			Sometimes	32.7		X
			Yes	60.1		
8	Is colostrum collected without disinfection of the teats prior to collection?	301	No	23.3		
			Sometimes	25.9	50:50	
			Yes	50.8		
9	Are heifer replacement calves fed with pooled colostrum?	302	No	26.5		
			Sometimes	27.8	X	
			Yes	45.7		
10	Are heifer replacement claves fed pooled surplus milk from healthy cows?	303	No	12.2		
			Sometimes	25.1	X	
			Yes	62.7		
11	Are heifer replacement calves fed milk from sick and mastitic cows?	304	No	40.5		
			Sometimes	33.9	X	
			Yes	25.7		
Manure management						
12	Is milk and feed area for calves contaminated with cow manure?	302	No	88.7		
			Sometimes	10.6	50:50	
			Yes	0.7		
13	Do calves have direct contact with cows and their manure prior to weaning?	300	No	78.3		
			Sometimes	13	50:50	
			Yes	8.7		
14	Do calves have access to pasture which has had cow slurry applied in the same season?	302	No	27.8		
			Sometimes	53.6	x	
			Yes	18.5		
15	Do heifers have direct contact with cows and their manure prior to entering milking herd?	302	No	21.9		
			Sometimes	40.7	50:50	
			Yes	37.4		
16	Is water and feed area for heifers contaminated with cow manure?	302	No	75.2		
			Sometimes	14.9	x	
			Yes	9.9		
17	Do heifers have access to pasture which has had cow slurry applied in the same season?	304	No	11.5		
			Sometimes	57.9	x	
			Yes	30.6		

*A subset of the population was re-surveyed to quantify the Sometimes responses. X indicates the response chosen by the majority of the subpopulation, 50:50 indicating an equal number choosing A or B.

### Descriptive analysis and herd classification

Hardcopy survey responses were entered into an online survey software package (http://www.surveymonkey.com) with electronic inputs being manually checked against hardcopy versions. Coded responses to each question were subsequently downloaded and Microsoft Excel (MS Office, Version 2010) used to organise the data, fix variables for directionality, and complete descriptive analysis.

Questionnaires were deemed suitable for analysis if greater than two thirds of survey questions were answered. Based on Irish Central Statistics Office [40] data, study herds were assigned to two geographical regions i.e. dairy dense (southern region) and non-dense (northern region), with herd calving-season categorised as spring-calving (i.e. ≥85% of the herd calved between January and March) and non-spring-calving (calving at other times of year). The livestock enterprise type was classified as dairy only or mixed-species livestock herds (i.e. herds that also contained beef cattle and/or sheep), with herd size categorised as small (31 to 65 cows), medium (66 to 99 cows), or large (>99 cows). The bioexclusion classification of each herd i.e. open (free movement of new purchases onto the farm) or closed (no introduction of new purchases onto the farm) was available from a parallel study as were a number of additional management factors [39].

### Statistical analysis

Chi-squared, logistic regression and correlation (Pearson and Spearman) analyses were completed using Stata data analysis and statistical software (Version 12). Prior to statistical analysis an initial model was created with ‘sometimes’ response options excluded. This allowed direct comparison between those answering definitively ‘yes’ or ‘no’ (Model 1). In the interest of completeness, survey response options were also dichotomised yielding two further datasets for analysis i.e. Model 2 = Y + S versus N and Model 3 = Y versus S + N. A total of five herd classification independent variables (i.e. region, calving-season, enterprise type, herd size, bioexclusion status) were used to examine key influences on JD risk variables. As a first step, a univariable (Pearson’s Chi-squared) analysis was completed. Independent variables recording P ≤ 0.15 were included in logistic regression models (1, 2 and 3). A manual backwards elimination with a forward step was applied to each model with significant variables (P ≤ 0.05 chosen as accepted significance level) retained in the final model. Pearson’s correlation was used to check for co-linearity across independent variables. Spearman’s rank correlation *(r*_*s*_*)* was performed to examine relationships between dependent variables (JD survey questions) with *r*_*s*_ values of >0.3 reported. Biosecurity variables were not statistically analysed.

## Results

### JD questionnaire descriptive and statistical analysis

A total of 306 farmers returned JD questionnaires suitable for analysis yielding a 98% response rate. Following exclusion of six questionnaires as incomplete, non-responders for individual JD questions ranged from two to nine participants. The majority of study herds (67%) were located in the dairy dense region of Ireland. Of the participating herds, 27% were categorised as small, 31% as medium, and 42% as large herd size. Similar to the national trend in the Republic of Ireland [41,42], spring-calving systems were operated by 87% of study herds, with 52% operating mixed livestock enterprises. A total of 54 herds (17.7%) were categorised as closed [39]. As results from Model 1 (Table 2) represented farmers that were definitive in responding either Yes or No to survey questions, this model is discussed in detail. Comparable associations, however, were observed in all three Models (Table 2 and Additional file 1).

**Table 2:** **Significant associations between independent and dependant variables (Model 1: Yes versus No)**

**Dependent variable**	**Odds ratio**	***P*** **value**	**Conf. interval (95%)**
Independent Variable			
**Is the CA overcrowded?**			
>99 cows *vs*. 66–99 cows	5.0	0.001	1.9, 13.0
>99 cows *vs*. 31–65 cows	7.8	<0.001	2.6, 23.6
**Do new born calves stay in CA for more than six hours?**			
31-65 cows *vs*. >99 cows	3.1	0.009	0.1, 0.8
Non-spring >99cows *vs*. spring 31–65 cows	28.1	0.016	1.9, 421.4
**Is the CA cleaned and bedded between every calving?**			
31-65 cows *vs*. >99 cows	3.6	0.001	0.1, 0.6
**Are heifer calves fed pooled colostrum?**			
66-99 cows *vs*. 31–65 cows	2.2	0.039	1.0, 4.5
>99 cows *vs*. 31–65 cows	4.8	<0.001	2.3, 9.9
>99 cows *vs*. 66–99 cows	2.2	0.024	1.1, 4.4
**Is colostrum collected without teat disinfection?**			
Mixed enterprise *vs*. Dairy only	1.8	0.049	0.3, 0.9
**Are heifer calves fed waste milk from sick cows?**			
Mixed enterprise *vs*. Dairy only	2.2	0.009	1.2, 3.9
**Do calves have direct contact with cows/ manure pre entering milking herd?**			
Non-dairy dense *vs*. Dairy dense	2.5	0.034	0.2, 0.9
**Do heifers have direct contact with cows/ manure pre entering milking herd?**			
>99 cows *vs*. 66–99 cows	2.5	0.019	1.2, 5.2
**Do heifers have access to pasture spread with cow slurry?**			
>99 cows *vs*. 66–99 cows	7.5	0.028	0.1, 0.9

*P* Value: Significant *P* <0.05. CA: calving area.

### Calving area (CA) variables

Only 3% of study farms avoided frequent use the CA for more than one calving at a time. Overcrowding of the CA, on at least an occasional basis, was reported by over 40% of respondents (having five or greater cows in the CA at any one time was cited in the questionnaire as an example of overcrowding following questionnaire piloting). Larger sized herds were more likely to overcrowd the CA compared to small (OR 7.8) or medium sized (OR 5.0) herds. Over two thirds of the participating farmers did not clean and bed the CA between calvings. Smaller herds however were more likely to engage in cleaning and bedding of the CA (OR 3.6) compared to larger herds. Sick cows were housed in the calving area by over half of all respondents.

### New-born calf management

Over 80% of farmers allowed a calf to remain with its dam in the CA for longer than six hours. Large non-spring calving herds were more likely than small spring calving herds to allow this occur (OR 28.1). Smaller herds, however, were more likely than larger herds to allow calves to remain in the CA for longer than six hours (OR 3.1). Unsurprisingly, a relatively strong relationship was highlighted between time spent in the CA area and allowing the calf to suckle the dam (*r*_*s*_ 0.5), although the vast majority of farmers (90%) allowed the calf to suckle the dam regardless of the amount of time spent together.

Approximately 70% of respondents pooled colostrum for feeding calves and almost 90% pooled milk for the same purpose. Larger herds were more likely to pool colostrum than smaller (OR4.8) or medium sized herds (OR2.2). Feeding calves with milk from sick or mastitic cows (waste milk) was practiced in almost 60% of herds on at least an occasional basis. This was more likely to occur in mixed enterprise herds as opposed to dairy only herds (OR 2.2). Mixed enterprise herds were also more likely to collect colostrum without teat disinfection (OR 1.8). Relationships existed between those farmers pooling milk and pooling colostrum *(r*_*s*_ 0.5), those feeding waste milk to calves and pooling milk for calf feeds (*r*_*s*_ 0.4), and also between those feeding waste milk and those pooling colostrum *(r*_*s*_ 0.3) (Table 3).

**Table 3:** **Spearman correlation values between dependent variables**

	**Q1**	**Q2**	**Q3**	**Q4**	**Q5**	**Q6**	**Q7**	**Q8**	**Q9**	**Q10**	**Q11**	**Q12**	**Q13**	**Q14**	**Q15**	**Q16**	**Q17**
Q1 Is the CA used for more than one calving?	1																
Q2 Is the CA overcrowded?	0.2	1															
Q3 Is the CA cleaned out between every calving?	0	**−0.3**	1														
Q4 CA used to house sick cows?	0	0.1	−0.1	1													
Q5 Cows have manure soiled legs and udders?	0.1	0.2	−0.1	0.2	1												
Q6 Calf in CA > 6 hours	0	−0.1	0.1	0	0.1	1											
Q7 Calf allowed to suck from the cow?	0	0.1	0.1	0.1	−0.1	**0.5**	1										
Q8 Colostrum collected without disinfection of the teats	0.1	0.2	−0.1	0.2	0.2	0.1	0	1									
Q9 Calves fed with pooled colostrum?	0	0.2	−0.2	0.2	0.1	0.1	0.1	**0.3**	1								
Q10 Calves fed with pooled milk?	0.1	0.1	−0.1	0.2	0.1	0.1	0.1	**0.3**	**0.5**	1							
Q11 Calves fed with waste milk?	0	0.1	−0.2	**0.3**	0.2	0.1	0	**0.3**	**0.3**	**0.4**	1						
Q12 Calf feed area contaminated with manure?	0.1	0.1	0	0.1	0.2	0.1	0.1	0.2	0.2	0.1	0.1	1					
Q13 Direct calf/ cow contact?	0.1	0.1	−0.1	0.1	0.2	0.1	0.1	0.1	0.1	0.1	0.1	0.2	1				
Q14 Calves have access to pasture which has had cow slurry applied?	0.1	0.1	−0.1	0.1	0.1	0.1	0.1	0.1	0.2	0.1	0.2	0.1	0.1	1			
Q15 Direct heifer cow contact?	0.2	0.2	−0.1	0.2	0.1	0.1	0.1	0.1	0.2	0.2	0.2	0.1	0.2	0.2	1		
Q16 Heifer feed area contaminated with manure?	0.1	0.2	0	**0.3**	**0.3**	0.1	0.1	0.2	0.2	0.1	0.1	**0.4**	0.2	0.1	**0.3**	1	
Q17 Heifers have access to pasture which has had cow slurry applied	0.2	0.1	−0.1	0.1	0.1	0.1	0.2	0.2	0.1	0.1	0.1	0.1	0.1	**0.5**	0.2	0.2	1

Correlations > 0.3 in bold. See Table 1 for entire list of questions.

### Hygiene and faeces management

Direct access between young calves and adult cows or their manure was prevented by the majority of survey participants (78.3%), however farms in non-dairy dense regions were over twice more likely to allow contact occur (OR 2.5) With regard to replacement heifers (>12 months), 78.1% of farmers allowed at least occasional direct heifer-cow contact to occur. Larger herds were more likely than medium sized herds to allow this heifer-cow contact to occur (OR 2.5). Over 70% of herds allowed young calves access to pasture which had slurry applied in the same grazing season, with almost 90% of participants allowing replacement heifers access to slurried pastures. Again larger herds were more likely than medium sized herds to allow heifers access such pasture (OR 7.5). A positive correlation existed between those herds allowing access of calves and heifers to potentially contaminated pastures (*r*_*s*_ 0.5). The majority of those surveyed prevented faecal contamination (from adult cows) of both young calf and replacement heifer feed areas and water troughs (88.7% and 75.2% respectively).

### Biosecurity questionnaire descriptive analysis

A total of 312 participants returned valid biosecurity questionnaires. Almost all study participants reported regularly inspecting farm boundaries (97.4%), with the majority also preventing access to watercourses passing through neighbouring farms (69.4%). Cattle trailers, water troughs, and oral drenching equipment were regularly cleaned by a large proportion of farmers, 81.7%, 74%, and 86.2% respectively. While almost 80% of farmers prevented mixing of different farm livestock species, less than 10% prevented nose to nose contact between different management age groups (i.e. cows, heifers, calves) on farm. The majority of farmers reported daily cleaning of walkways and collecting yards and also annual disinfection of all cattle housing. Only 36.2%, however, reported cleaning individual calf pens between successive calves. Isolation of sick animals was reported by nearly 90%, and in general, importation of colostrum and various manure types was avoided with over 90% of study farmers not engaging in such practices. Additional biosecurity practices are included in Figure 1 in order of the number of farmers implementing each measure.

## Discussion

Closure of transmission routes [43] and improved calf management [8] are essential elements of MAP control at farm level. The aim of this survey was to document JD-related management practices utilised on Irish dairy farms, thereby identifying target areas for improvement in future studies and control programmes. Questions were designed to highlight management practices that have been associated with a risk of MAP transmission in the literature. In general it was found that management practices currently being implemented by Irish dairy farmers pose a high risk of MAP infection, with larger herd sizes more likely to engage in hazardous practices for MAP transmission.

Previous international studies reported an increased risk of MAP transmission in herds where more than one cow was allowed in the calving area [17,18], and in herds that do not routinely clean the CA between calving’s [21] . The Irish system of dairy production is an extensive, pasture-based system, with cows grazing grass outdoors for prolonged periods of lactation [44]. This combined with a relatively low average herd size compared to other countries [42,45,46], might be expected to lead to a less intensive calving system with minimal CA overcrowding and good hygiene. The results presented in the current study, however, highlight that this system does not necessarily lead to optimal CA management. Pasture-based systems must operate within the constraints of the grass-growing season, and as such, a highly seasonal calving pattern is adopted [41,47]. As compact-calving herds only experience approximately one month of concentrated calving [42], it is possible that Irish farmers invest in the infrastructural capacity to deal with herd average calving rate, as opposed to maximal calving rate, leading to overcrowding of the CA at certain times of the calving season. The sub-optimal levels of CA cleaning between calving’s, and the CAs frequent use, is also potentially reflective of inadequate time and infrastructural resources provided to manage the period of intensive calving in spring. The fact that larger herds are less likely than smaller herds to clean (OR 0.27), and more likely to overcrowd (OR 7.8) the CA provides further support for this theory, with larger herds having more intensive calving seasons. The seasonal calving system operated in Ireland, therefore, could potentially lead to increased transmission of MAP by bringing about sub-optimal management of the CA. Education is therefore required to highlight the importance of optimal calving management, and availability of adequate resources (especially at peak calving season), and its contributing role in achieving effective control of JD.

Regarding use of the CA for isolation and treatment of sick cows, the proportion of farmers engaging in this practice in Ireland is similar to that reported internationally (approximately 50%) [48-50]. This may again reflect increased efficiencies being sought by farmers through assigning multiple uses to existing farm infrastructure. While dual use of the CA (for both calving and hospitalisation) may be considered optimal usage of this infrastructural resource, it is placing herd-cohorts at undue risk of pathogen exposure [51]. Indeed, Norton S, Heuer C and Jackson R [52] highlighted an increased risk of MAP incidence in a herd when calves are raised in an area used for cow hospitalisation. As calves are born with naïve immune systems [53], use of the CA for cow hospitalisation does not present a rational use of farm infrastructure in regard to disease prevention and control.

Additional management practices commonly utilised on dairy farms to achieve greater resource efficiency include pooling of colostrum, pooling milk, and use of waste milk as a calf feed [54]. Colostrum is pooled to potentially provide passive immunity from vaccinated cows [55,56] and to ease availability of adequate volumes of colostrum during periods of peak calving, with pooling of milk facilitating group feeding of calves. Pooling of calf feeds are highly attractive for farmers in terms of resource efficiency which may account for their extensive use on Irish farms. Additionally, Gleeson D, O’Brien B and O’Donovan K [57] showed that calf management labour-saving practises were more likely to be used as herd size increases. It is perhaps not surprising, therefore, that large (OR4.8) and medium (OR 2.2) sized herds in the current study were more likely to engage in the practice of pooling colostrum compared to smaller sized herds. This may also underpin the widely acknowledged increased risk of larger herds testing positive for MAP [23,38] as pooling of both colostrum and milk is also associated with increased risk of MAP transmission within a herd [14,58].

Waste milk may be perceived as a useful resource on dairy farms, with farmers reluctant to discard it. Waste milk can be regarded as a cost saving measure rather than using saleable milk or milk replacer as calf feeds [54] Although such feed management practices may be perceived as being resource efficient, feeding of waste milk has been associated with risk of exposure to a number of pathogens [59], including MAP [16]. More specifically, a univariate analysis completed by Barrett D, Mee J, Mullowney P, Good M, McGrath G, Clegg T and More S [38] examining risk factors for testing MAP faecal culture positive, found a significant association between pooling colostrum, feeding waste milk and testing MAP culture positive. The practice of feeding waste milk is not unique to Irish dairy farmers, however, with the current study recording a slightly lower prevalence of this practice compared to UK and Australian farmers [60,61]. The correlation (*r*_*s*_0.3) between farmers in the current study that use the CA for housing sick animals, and feed waste milk to heifer calves, again supports a trend amongst farmers in seeking, and using, resource efficient management methods regardless of potential disease consequences. A balance therefore needs to be sought and promoted amongst farmers to allow practical and cost-efficient rearing of dairy calves without increasing exposure to potential harmful pathogens.

Opinions of veterinary experts and practitioners reported by Sayers R, Good M and Sayers G [62] highlights avoiding slurry importation, the up keep of farm boundaries and maintaining accurate disease records as key elements in farm biosecurity, all of which the majority of the current study participants conducted, indicating a level of good biosecurity practice implementation on farm. Veterinary experts however, ranked farmer understanding of a disease second only to maintenance of a closed herd when promoting optimum farm biosecurity [62]. While many of the JD-associated management practices used on farm appear to be resource/efficiency driven, their implementation may be due to a lack of fundamental understanding of the JD risk involved when adopting certain practices. Sayers R, Sayers G, Mee J, Good M, Bermingham M, Grant J and Dillon P [27] have reported farmers acknowledge the importance of biosecurity, but that lack of information may prevent improvement of biosecurity practices. The findings of the present study highlights the importance of ensuring farmers evaluate labour and cost saving management routines prior to their introduction on farms and are fully educated regarding potential disease transmission risks associated with such efficiencies. As this study has identified comparable management practices reported in international studies, the opportunity exists to examine how countries with more established control programmes tackled similar management issues to help limit MAP transmission.

A possible weakness of the current study is the use of self-reported responses, as evidenced by 43.3% of those surveyed reporting cows not to have manure soiled legs or udders. This weakness indeed highlights the need for independent on farm risk evaluation. The RAMP by AHI now provides such an independent verification and will prove extremely useful in tracking the progress of Ireland’s JD control programme. In general, however, it can be concluded from this current study that a high proportion of Irish dairy farmers are engaging in practices associated with increased risk of MAP transmission. Based on existing studies, however, the prevalence of JD in Ireland, compares favourably with other milk producing nations [31,32,63]. The relatively small size of Irish dairy herds (average herd size 60 cows), compared to other intensive dairy systems (e.g. average herd size US:120 cows; average herd size New Zealand: 393 cows) [42,45,46] may contribute to the lower recorded prevalence, larger herds being at higher risk of contracting JD [23,24,38] As Irish farmers intend to expand their dairy herds post-2015 due to the abolition of milk quotas (restriction on milk production) [64], the overall risk of contracting JD in Ireland may increase. Additionally, as it is unlikely that all expanding herds will achieve required cow numbers within the breeding capacity of their own herds, purchase of dairy stock is likely to increase further. With an already low level of closed herds operating in Ireland currently, a further increase in the purchase and movement of livestock may exacerbate the risk of MAP transmission [28]. Positively, however, Sayers R, Sayers G, Mee J, Good M, Bermingham M, Grant J and Dillon P [27] have highlighted that Irish dairy farmers with larger herds are more likely to voluntarily join a health scheme, making establishment of AHI’s JD programme a timely intervention.

## Conclusion

Many management practices associated with risk of MAP transmission were commonly applied on Irish dairy farms. Larger herds were more likely to engage in high risk practices for JD transmission. Control programmes should incorporate educational tools outlining the pathogenesis and transmission of MAP to highlight the risks associated with implementing certain labour-saving measures with regard to JD transmission. Programmes would also benefit from promoting evaluation of management practices, for impacts on disease control, prior to their introduction on-farm.

### Endnotes

http://www.animalhealthireland.ie

http://www.myhealthyherd.com

## Additional file

Additional file 1: Table S4a and b. Significant associations between independent and dependant variables (Model 2 and Model 3).
