# Supplementary material for: A survey of management practices on Irish dairy farms with emphasis on risk factors for Johne’s disease transmission
Source: Ir Vet J. 2014 Dec 24;67(1):27. doi: 10.1186/s13620-014-0027-9 (PMC4300563; doi:10.1186/s13620-014-0027-9)
Supplement: Additional file 1: Table S4a and b. — Significant associations between independent and dependant variables (Model 2 and Model 3). [file 13620_2014_27_MOESM1_ESM.docx]

**Table 4a:** Significant associations between independent and dependant variables

(**Model 2**: Yes + Sometimes versus No)

| **Dependent Variable** | **Odds Ratio** | ***P* Value** | **Conf. Interval (95%)** |
| --- | --- | --- | --- |
| Independent Variable |  |  |  |
| **Is the CA overcrowded?** |  |  |  |
| 66-99 cows *vs*. 31-65 cows | 2.24 | 0.017 | 1.15, 4.3 |
| >99 cows *vs*. 31-65 cows | 3.78 | <0.001 | 2.01, 7.1 |
| Dairy only, 31-65 cows *vs*. Mixed enterprise, 66-99 cows | 7.14 | 0.027 | 0.02, 0.8 |
| Dairy only, 31-65 cows *vs*. Mixed enterprise, >99 cows | 5.88 | 0.039 | 0.03, 0.9 |
| **Is the CA used to house sick cows?** |  |  |  |
| Dairy dense *vs*. not | 2.37 | 0.001 | 1.43, 3.9 |
| **Do new born calves stay in CA for more than six hours?** | | |  |
| Non-dense, non-spring *vs* Dairy dense, spring calving | 11.62 | 0.043 | 0.01, 0.92 |
| **Is the CA cleaned and bedded between every calving?** | | |  |
| 31-65 cows *vs*. >99 cows | 2.12 | 0.022 | 0.25, 0.9 |
| Non-spring *vs*. spring | 2.41 | 0.046 | 1.01, 5.7 |
| **Are heifer calves fed pooled colostrum?** | | |  |
| 66-99 cows *vs*. 31-65 cows | 2.20 | 0.013 | 1.19, 4.4 |
| >99 cows *vs*. 31-65 cows | 3.55 | <0.001 | 1.87, 6.8 |
| Mixed, non-spring *vs*. dairy only, spring calving | 6.29 | 0.028 | 1.22, 32.4 |
| **Are heifer calves fed pooled milk?** | | |  |
| Closed *vs*. Open | 8.40 | 0.039 | 0.02, 0.9 |
| **Are heifer calves fed waste milk from sick cows?** | | |  |
| Dairy dense, > 99 cows *vs*. non dense, 31-65 cows | 3.87 | 0.030 | 1.14, 13.2 |
| Mixed enterprise *vs*. Dairy only | 2.37 | <0.001 | 1.45, 3.8 |
| **Do calves have access to pasture spread with cow slurry?** | | |  |
| Open *vs*. Closed | 2.07 | 0.027 | 1.09, 3.9 |
| Closed, dairy only *vs*. Open, mixed enterprise | 13.15 | 0.001 | 0.02, 0.4 |
|  |  |  |  |

*P* Value: Significant *P* <0.05 CA: calving area

**Table 4b:** Significant associations between independent and dependant variables

(**Model 3:** Yes versus Sometimes + No)

| **Dependent Variable** | **Odds Ratio** | ***P* Value** | **Conf. Interval (95%)** |
| --- | --- | --- | --- |
| Independent Variable |  |  |  |
| **Is the CA frequently used for more than one calving at any one time?** | | |  |
| Spring *vs*. Non- spring | 2.38 | 0.014 | 1.2, 2.9 |
| Mixed enterprise *vs*. dairy only | 1.72 | 0.022 | 0.2, 0.8 |
| **Is the CA overcrowded?** | | |  |
| Dairy dense 66-99 cows *vs*. non dense 31-65 cows | 29.27 | 0.031 | 1.35, 632.6 |
| Dairy dense > 99 cows *vs*. non dense 31- 65 cows | 13.22 | 0.032 | 1.25, 139.9 |
| **Do new born calves stay in CA for more than six hours?** | | |  |
| 31- 65 cows *vs*. >99 cows | 2.70 | 0.001 | 0.20, 0.7 |
| Open *vs*. Closed | 1.91 | 0.05 | 0.99, 3.6 |
| **Is the CA cleaned and bedded between every calving?** | | |  |
| 31- 65 cows *vs*. >99 cows | 2.08 | 0.019 | 0.25, 0.9 |
| **Are heifer calves fed pooled colostrum?** | | |  |
| Dairy dense *vs*. not | 1.66 | 0.047 | 1.19, 4.4 |
| >99 cows *vs*. 31-65 cows | 2.70 | 0.001 | 1.48, 4.9 |
| **Do calves have direct access to cows/ manure before weaning** | | |  |
| Dairy dense, 66-99 cows *vs*. non-dense, 31-65cows | 40.0 | 0.018 | 1.89, 843.4 |
| **Are heifer feed and water contaminated with cow manure**? | | |  |
| Mixed enterprise *vs*. dairy only | 2.53 | 0.032 | 1.08, 5.9 |
| **Do heifers have access to pasture spread with cow slurry**? | | |  |
| Spring *vs*. Non- spring | 2.70 | 0.034 | 0.15, 0.9 |

*P* Value: Significant *P* <0.05. CA: calving area.
